# Understanding the Emotional Intelligence Discourse on Social Media: Insights from the Analysis of Twitter

**DOI:** 10.3390/jintelligence9040056

**Published:** 2021-11-24

**Authors:** Shardul Shankar, Vijayshri Tewari

**Affiliations:** Department of Management Studies, Indian Institute of Information Technology, Allahabad 211015, India; vijayshri@iiita.ac.in

**Keywords:** emotional intelligence, Twitter, social media, emotional analysis, sentiment analysis, network analytics

## Abstract

Social networks have created an information diffusion corpus that provides users with an environment where they can express their views, form a community, and discuss topics of similar or dissimilar interests. Even though there has been an increasingly rising demand for conducting an emotional analysis of the users on social media platforms, the field of emotional intelligence (EI) has been rather slow in exploiting the enormous potential that social media can play in the research and practice of the framework. This study, thus, tried to examine the role that the microblogging platform Twitter plays in enhancing the understanding of the EI community by building on the Twitter Analytics framework of Natural Language Processing to further develop the insights of EI research and practice. An analysis was conducted on 53,361 tweets extracted using the hashtag emotional intelligence through descriptive analytics (DA), content analytics (CA), and network analytics (NA). The findings indicated that emotional intelligence tweets are used mostly by speakers, psychologists (or other medical professionals), and business organizations, among others. They use it for information dissemination, communication with stakeholders, and hiring. These tweets carry strong positive sentiments and sparse connectedness. The findings present insights into the use of social media for understanding emotional intelligence.

## 1. Introduction

Of late, cyberspace and social media have become increasingly adjacently parallel to the physical world, especially when it comes to conversations or discourse (Wei et al. 2019). Social networks have allowed for easier connections with people right from a person’s couch and they have increased the convenience of people’s daily lives, but they have also opened people’s minds regarding security and privacy concerns that come with the ease of use of these platforms. The industry–academic community has grown significantly interested in studying the potential use that these social networks’ data could provide to businesses or the research community. The large quantities of data generated (commonly called “big data”) allow researchers to extract information from these data, create new insights into the different domains, and understand users’ characteristics, behavior, and decision-making patterns. Literature regarding social media data has studied the demographical characteristics of the users (Jurgens et al. 2015), the users’ psychological traits expressed through social networks (Burrus et al. 2012; Chen et al. 2017; Kosinski et al. 2014; Xu et al. 2008), stock price predictions (Huang and Liu 2020; Tsui 2017), epidemics and pandemics (Cinelli et al. 2020; Zhao et al. 2020; Kadam and Atre 2020; Gao et al. 2020), elections (Jaidka et al. 2019; Ceron et al. 2016; Ferrara 2020), brand management (Jin 2012; So et al. 2018), information diffusion (Stieglitz and Dang-Xuan 2013; Kushwaha et al. 2020), public opinion (Gorodnichenko et al. 2018; Ford et al. 2019; Hickerson and Kothari 2017), and healthcare (Terry 2009; Courtney et al. 2013; Pizzuti et al. 2020).

The domain of emotional intelligence (EI) has been relatively dawdling when it comes to utilizing the potential that social network platforms and their data can play in predicting the users’ emotional intelligence. Even though there has been growth in recent times in using social media to understand the facets of EI (Menon and Rahulnath 2016; Hornung et al. 2018; Madaan et al. 2020), these studies have relied on traditional methods of data collection and self-reporting techniques (e.g., survey techniques or interviews) and statistical techniques (e.g., moderation and mediation analyses). Nevertheless, there has been a recent shift in using big data in the area of EI (Wei et al. 2019; Abkenar et al. 2020). Even though organizations are perfectly capable of generating big data on their employees, social media platforms (e.g., Facebook, Twitter, YouTube, Reddit, etc.) make the vast majority of contributions towards these big data.

A study (Cecere 2012) encompassing leaders and industry heads found out that one-third of modern organizations are using big data to understand their employees. The organizations also believed that the traditional means of generating big data are competent for effectively managing behavioral and transactional data, but were uncertain about using social media data for generating business intelligence. This was echoed in other industry surveys (Natoli 2013; Mann 2014), where business decisions were being effectively leveraged by these organizations, but less than 1% explained that they were able to use social media for human resource planning and organizational behavior. They believed that social media would transform organizational psychology by effectively studying the emotional intelligence of their employees, but a majority of these organizations had no idea where to even start looking for it.

In our study, we concentrated our efforts on one particular social media giant, Twitter (Twitter 2021). This specific social media platform was chosen over its competitors because of its microblogging capability and the fact that it is arguably the fastest growing social media platform there is (Sharma et al. 2017). Twitter is increasingly used by its users for multiple reasons, including discussing mental issues, sharing news and personal feelings, or expressing opinions about political and ideological themes in a brand/organization/celebrity discussion (Chae 2015). The industrial community or researchers can access Twitter data through their APIs (application programming interface) to analyze the data for various domains of study.

This study was carried out to build upon the understanding of social media in the context of emotional intelligence. This is achieved by using natural language processing techniques to analyze tweets containing contexts of emotional intelligence, and the associated people or users who were discussing EI on the Twitter platform, to develop insights into EI practices and research and the potential role that Twitter can play in this. This thought was echoed in studies on industry experts and organizations (Chae 2015; Cecere 2012; Zhang et al. 2019), as there is a clear lack of an insightful understanding of the concept, and there is very little literature to support this understanding.

Even though there have been a lot of proposed frameworks for understanding emotion expression, textual emotion, and the underpinnings of emotions in real-time data, there is a significant lack in the literature when it comes to creating a framework that understands the contextual understandings of the discourse of emotional intelligence, especially in social media (Israelashvili et al. 2021). The motivation of this study was to propose a framework that can be used to better understand how emotional intelligence is discussed in social media and social networks, and how these discussions are driving understandings of the emotional psyche. This study would thus try to create a framework on which future researchers and industries can build to generate knowledge from Twitter data in the EI community.

Chae (2015) described Twitter Analytics (TA) as an analytical technique for analyzing Twitter data for a research outcome. They stated that TA is a combination of three analytics—descriptive analytics (DA), content analytics (CA), and network analytics (NA). We have tried to modify this framework to extract the information pertaining to emotional intelligence. These three analytical techniques focus on multiple magnitudes of Twitter data. The collected tweets and metadata covered the discussions of individual users, professionals, and organizations in terms of the concept of EI. Specifically, the findings of the analyses have tried to answer these research questions:

(1) Are there any patterns in the characteristics of the information diffusion of emotional intelligence tweets?
(2) Are there any dominant topics, content, or discussions that are being shared on Twitter regarding emotional intelligence?
(3) Are there any patterns in the characteristics of the Twitter users who indulged in dialogues on emotional intelligence?
(4) Are there any patterns in the sentiments of the emotional intelligence tweets, and what are the tweet contents that contain sentiments of emotional intelligence?

Accordingly, the research is divided into sections as follows: Section 2 presents the present literature about the use of Twitter in multi-dimensional domains, Section 3 presents the data collection and pre-processing methodologies, Section 4 discusses the framework of Twitter Analytics, Section 5 provides an analysis of the collected tweets using TA, and the final sections conclude the study by presenting the research implications, limitations, and scope for future research.

## 2. Literature Review 

Since its inception in 2006, Twitter has become one of the biggest microblogging websites, with 500 million daily and 200 billion yearly tweets (Twitter 2020), and 150 million monetizable users (Tankovska 2021). A study found out that over 75% of Fortune 500 companies have an active Twitter account, for their corporates and their brands (Malhotra et al. 2012). It has become one of the fastest information dissemination tools that allow for discussions, conversations, and even the spread of information that is true or false, making it one of the strongest assets for anyone with a voice.

A tweet (Vega et al. 2010), which is Twitter’s shared content, contains 280 characters, through which the users share their opinions and have real-time conversations. A tweet can be one of three types: an original tweet, a retweet, or a reply (Chae 2015). All of these messages can be traced manually or by using Twitter’s application programming interface (API). A popular tweet usually gets a status of “trending”, which helps for easier reach and conversations with followers (Aiello et al. 2013).

Due to its increasing popularity, Twitter is being used in varied domains of practical and academic research, including stock market forecasting and predictions (Bollen et al. 2011; Sóti et al. 2020), brand management (Zimbra et al. 2016; Lalicic et al. 2020), crisis management (Kersten and Klan 2020; Wang et al. 2021), healthcare (Alotaibi et al. 2020; Masip et al. 2020; Talbot et al. 2021), finance (Souza et al. 2015; Mao et al. 2011), information technology and information systems (Castillo et al. 2011; Ruz et al. 2020), supply chain management (Chae 2015; O’leary 2011), and psychology (Bogen et al. 2020; Dodds et al. 2011).

When talking about emotions and emotional intelligence, the domains of psychology, philosophy, sociology, organizational behavior, etc. have been extensively researched over a long time (Sailunaz and Alhajj 2019; Bryan and Mayer 2021). Initially, it was a part of biological evaluation, but with time, neuroscience has opened avenues for evaluating emotions as a socio-cognitive function (Tago and Jin 2018).

This shift in understanding the influence that emotional intelligence has on the limbic as well as neo-cortex systems, thereby creating a function of the neural system, has allowed the extraction mechanisms to become diverse—from primary and secondary data to more comprehensive experimental, experiential, and real-time big data and natural language processing (Tellez et al. 2017; Israelashvili et al. 2021). 

Many researchers have proposed their own frameworks for evaluating textual emotions (Binali et al. 2010; Jain et al. 2017) but these frameworks have a high presence of linguistic and methodological limitations (Sailunaz and Alhajj 2019). Moreover, these studies have focused primarily on understanding emotional triggers and have skimmed over the parameters of emotional regulation and intelligence.

This study also fights the criticism of previous studies (Hall and Mast 2007) that the verbal components of emotional discourse are usually missing in the evaluation of emotional intelligence. To address these issues, there have been several studies that have used real-time data to understand the underpinnings of the concept of emotion (Paul and Sui 2019; Suhasini and Srinivasu 2020; Lim and Birney 2021; İŞ and Tuncer 2018). 

Yet, despite the recent increase in the interest in using Twitter as a platform to study the domain of emotions, studies in the area of emotional intelligence are very scarce. One exception (Kumar and Devi 2020) used EI to study the perceptions of political parties. The objective of the present study was to identify the relationship between the tweet contents of emotional intelligence tweets among users, professionals, and organizations. The findings would contribute to the impact that Twitter has when it comes to understanding emotional intelligence in life and the workplace.

## 3. Data

The data collected for the proposed framework required the effort of manual classification of the tweets for the analysis of the extracted tweets. The initial extraction was performed using keywords such as “emotional intelligence”, “ei”, “eq” etc. and this gave us the understanding that #emotionalintelligence was the most prevalent hashtag that could be used in the study. The tweets were collected from 14th February 2021 to 6th March 2021, and which included 53,361 emotional intelligence tweets and their content.

The study was conducted using Python (version 3.8.7) using the Twitter API and the tweepy package of Python via Twitter. The tweets collected were public tweets, and private tweets were excluded from the collection (Gokulakrishnan et al. 2012). The privacy of the users was also maintained in the process, as the personal or private information of the users was removed from the analysis. A summary of the dataset is shown in Table 1.

### Data Pre-Processing

The tweets of any user at any given time are usually of three types: textual tweets, visual tweets, and auditory tweets. To make the information in them useful, a great amount of data cleaning was required, also known as data pre-processing. This was achieved by creating data tokens and using only the textual tweets, according to the process explained in Angiani et al. (2016) and Gokulakrishnan et al. (2012). The steps involved in data pre-processing were:

- Fixing grammatical, spelling, and punctuation errors;
- Fixing slang, acronyms, and colloquialisms;
- Removing numbers and digits;
- Removing exercising, gym, and workout data;
- Removing URLs by searching for http/https/www and removing the following text;
- Removing contractions and negations;
- Removing emojis and emoticons;
- Removing non-ASCII characters (including non-UTF-8 Unicode);
- Removing stop-words and extra spaces;
- Converting all the text to lower case;
- Stemming and lemmatizing the words

## 4. Framework for Twitter Text Analytics

**Figure 1:**
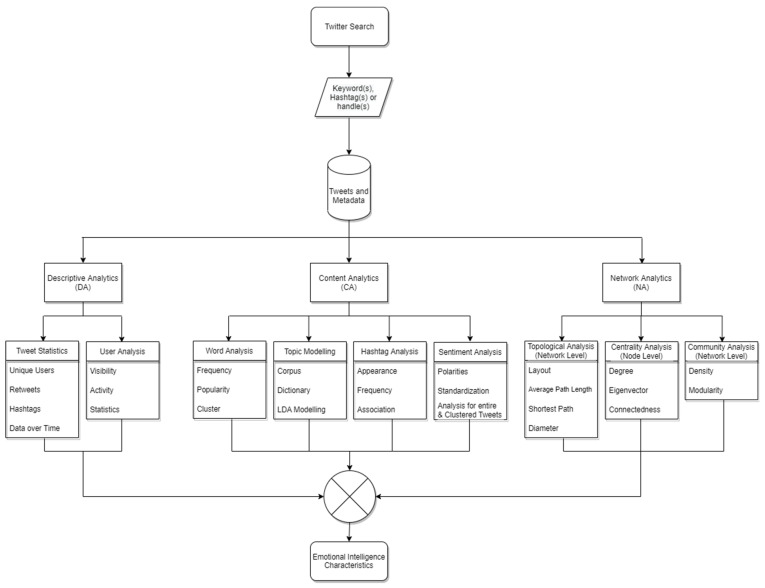
Research process framework for Twitter Analytics.

While data collections from social media platforms such as Twitter rely on APIs, the analysis is usually challenging, as the data have a lot of noise, are unstructured, and are substantially enlarged and enriched (Doldor et al. 2019) in comparison with their traditional counterparts. An analytical framework is also not readily available (Zeng et al. 2010), and hence, a framework encompassing the methods that extract and evaluate information from the data is required. The framework used in this study was initially developed in a study by Chae (2015) to analyze the Twitter dataset of supply chain tweets, which has been modified to evaluate the discourse on emotional intelligence. This framework has three analytics: descriptive analytics, content analytics, and network analytics. Figure 1 presents the relevant metrics of the analytical processes.

### 4.1. Descriptive Analytics (DA)

In this process, we focused mainly on the descriptive statistics of the dataset. The descriptive metrics and the user metrics gave us direction into other user-related information that was used in the content and network analytics. 

### 4.2. Content Analytics (CA)

The data collected were primarily unstructured in nature, and hence, natural language processing (NLP) was used to pre-process, format, and transform the data for word analysis, topic modeling, and sentiment analysis.

### 4.3. Network Analytics (NA)

With the help of the data and text obtained through the above processes, a network model was created using the GUI tool Gephi (Bastian et al. 2009). The nodes were the Twitter users and the edges were the relationships between these users.

## 5. Results

### 5.1. Descriptive Analytics

Descriptive analytics of the data from social media platforms is the initial building block for analyzing the social media data, not only for businesses but also researchers. DA was performed using the Python package Gensim (Cao et al. 2009) by using the understandings developed by Bruns and Burgess (2011), along with other statistical techniques. With the help of Python and its package, information about the users and tweets were extracted, and statistical techniques were used to visualize the statistics of the data.

#### 5.1.1. Tweet Statistics

Out of 53,361 tweets, unique users accounted for 41.36% (22,895), retweets accounted for 28.29% (15,096), and unique mentions accounted for 7.33% (4056), respectively. In total, 11,910 hashtags were used in these tweets, covering the four important factors of a traditional emotional intelligence model (motivation, self-awareness, empathy, etc.).

#### 5.1.2. User Analysis

**Figure 2:**
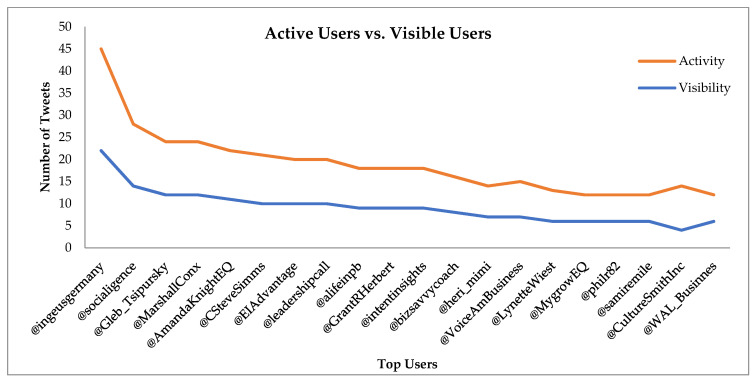
Most active vs. visible users.

There were 22,895 unique users in the dataset, indicating that every user, on average, sent 2.33 tweets, 1.52 retweets, and 0.81 mentions. Active users were calculated by using the formula (tweets + retweets + mentions), and visible users by the formula (retweets + mentions received). Figure 2 shows the active users and the visible users. The figure shows that the most active users were also the most visible users, which was expected. One important finding was that amongst the most active and visible users were the speakers that talk about motivation, emotional intelligence, and other soft skill topics.

### 5.2. Content Analytics

#### 5.2.1. Word Analysis

The most popular words found in the tweets were motivation (49,530 times in tweet texts), inspiration (13,383 times), empathy (2872 times), self-belief (1740 times), self-love (1566 times), care (1325 times), inspire (1328 times), emotional intelligence (1200 times), and self-care (1077 times), among others. 

#### 5.2.2. Topic Modeling

To further classify the clustering of the words, we used the topic modeling technique using Python’s Gensim package by creating a corpus and dictionary, according to the algorithm of Cao et al. (2009), and used these as inputs in LDA modeling. We were able to create four distinct topics with eight words in each topic. Table 2 presents the topic modeling outcomes from the word analysis.

#### 5.2.3. Hashtag Analysis

In total, 45,859 hashtags were found in the tweets, with the total occurrence of these hashtags being 447,747 times. The most popular hashtags were *#motivation*, *#inspiration*, *#emotionalintelligence*, *#success*, *#goals*, *#empathy*, *#positivity*, *#happiness*, *#mindfulness*, *#selflove*, *#wisdom*, *#believe*, *#training*, and *#selfcare*, including others. This showed that there were, on average, 8.39 hashtags per tweet, and the top three hashtags accounted for 15.74% (70,476 times) of the overall hashtag appearance in the tweets.

#### 5.2.4. Sentiment Analysis

Sentiment analysis was mostly done using the Python package Textblob (Zhang et al. 2018), and the tweets were categorized into three major polarities, i.e., positive, negative, and neutral. Table 3 presents the percentages of the polarities from the sentiment analysis of our tweet data. The overwhelming majority of emotions of these tweets were of positive sentiment, with the smallest percentage for positive sentiment being *self-awareness*, with 88.53%. Neutral sentiments were also higher than negative sentiments in our topics, indicating that when the sentiments were not positive, they were tending towards neutral sentiments. Negative sentiments regarding the topics were very meager, with the largest percentage being 2.95%, attributed to self-awareness.

**Figure 3:**
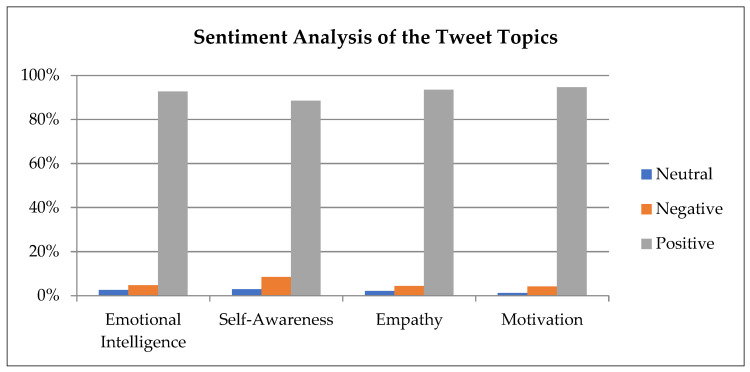
Tabular representation of the sentiment analysis of the tweet topics (in percentages).

To visually understand the sentiment analysis of the tweet, the first step was to create a tabulated representation of the percentage sentiment analysis of the tweet topics. Figure 3 shows a graphical representation of the percentage sentiment analysis of the tweet topics.

**Figure 4:**
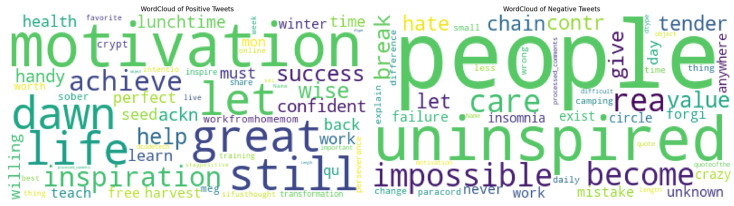
Word cloud of the sentiments of tweet topics.

Next, word clouds of the positive and negative sentiments were created. This was also achieved using the Textblob package of Python. Figure 4 shows the word clouds of the two sentiments according to their frequencies.

It can be seen that *motivation*, *great*, *life*, and *dawn* were the most frequently occurring positive words, and *people*, *uninspired*, *impossible*, and *insomnia* were the most frequently occurring negative words.

**Figure 5:**
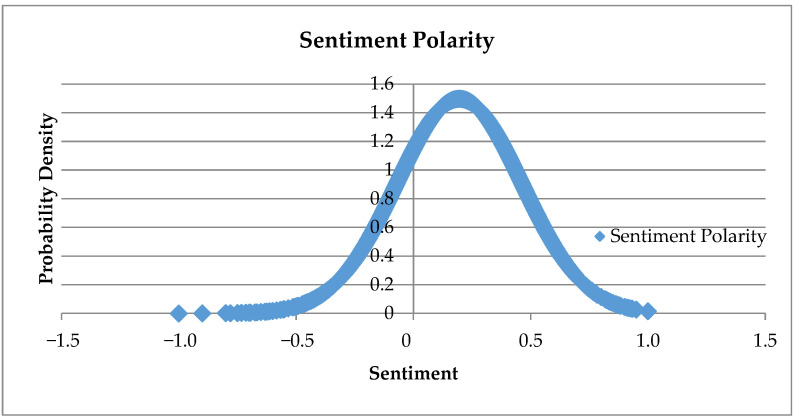
Standard normal distribution of the sentiment polarities of the topics.

Standardization of these sentiments [−1, 0, 1] was achieved using the SentiStrength package of Python (Thelwall et al. 2011). Even after standardizing the sentiment polarities of the topics, the normal distribution of these sentiments was pushed towards the positive side of the distribution. Figure 5 shows the normal distribution of the sentiment polarities of the dataset.

It can be seen from the figure that even though the distribution of sentiment polarity is normally distributed, most of the values of the curve are in the positive region of the curve.

**Figure 6:**
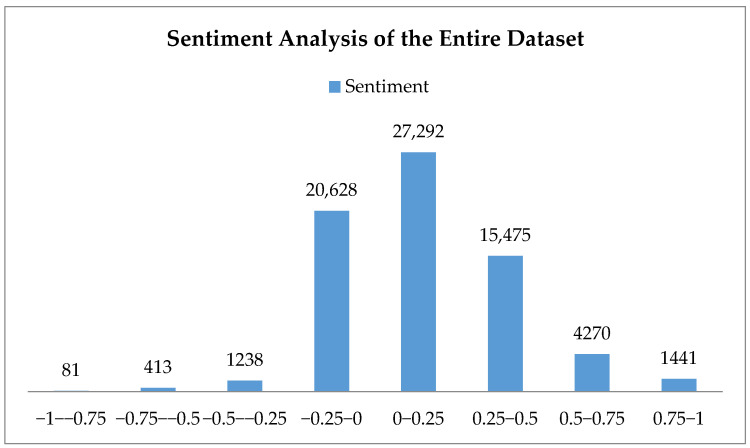
Sentiment analysis at the dataset level of the entire dataset.

A similar finding was observed when the entire dataset of the analysis was tested for sentiment analysis without the topics. Figure 6 shows the sentiment analysis of the entire dataset at the non-topical level. There was an obvious skew of the sentiment polarities towards the positive sentiment, with 68.44% of all the tweets ranging from 0 to +1, indicating an overall positive sentiment within the dataset.

### 5.3. Network Analytics

**Figure 7:**
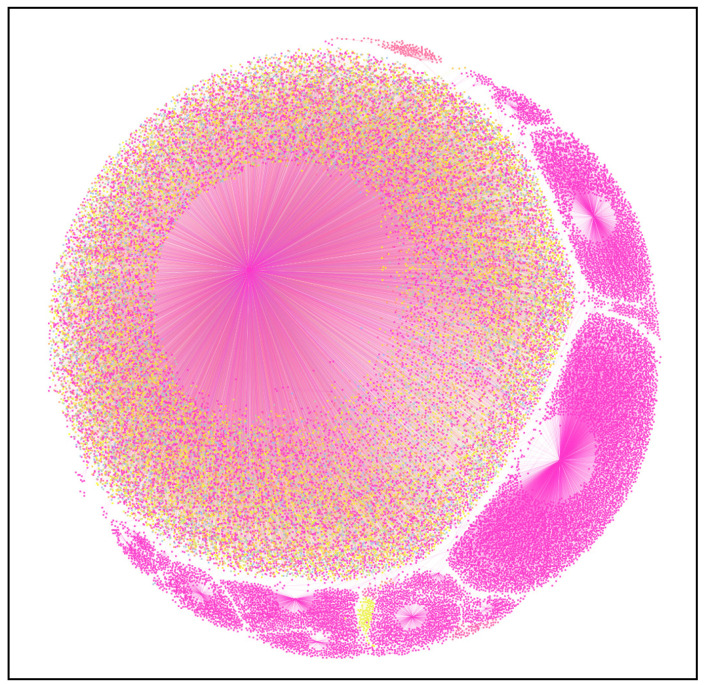
Network diagram of “emotional intelligence” in tweet data.

A topological social network was created to understand the node and network-level metrics of the user data. The network graph was created using the open-source software known as Gephi (Bastian et al. 2009). The nodes of the graph were the users that had interactions with each other using emotional intelligence as a content topic, and the edges were the mentions that the users gave or received using the @mentions process (Chae 2015). There were 4058 nodes and 217 edges. The network diameter was 5, indicating that the network was tight but sparse. Figure 7 shows the network graph of the distribution. The average path length was 2.405, which indicates that all the nodes were, on average, at least three nodes away from each other. This is consistent with the typologies of other networks studied in the domains of emotions (Watts 2004; Moeller et al. 2018).

To figure out the node connectedness, the in-degree of all the nodes was calculated to figure out the nodes’ popularity. The findings showed that speakers (@DriverClassics, @gvhawtin), doctors (@Gleb_Tsipursky, @denisemose), and businesses (@i_GotQ, @MotivationBytes) were amongst the most popular, with high tweets and mentions. Table 4 presents a tabulation of high in-degree users.

The graph also showed that the connectedness had 5% representation on average, further explaining that the network is sparse, with the largest community having 6% of total user representation, with the smallest community having 4% of user representation.

## 6. Discussion

The study was conducted on 53,361 tweets to examine the characteristics of the emotional intelligence tweets and the users discussing the topic. In the preliminary secondary analysis of the topic, it was clear that the literature in this domain is somewhat scarce, as multiple articles have discussed emotions in Twitter users and their tweets (Wang and Pal 2015; Dale et al. 2020), but there is relatively negligible research covering emotional intelligence in the Twitter users (Zhang et al. 2019). This study was thus carried out to create a branch of findings that can be used to add to the existing literature on the study of emotional intelligence. 

The rate at which people talk about emotional intelligence on Twitter was contrary to what was expected, as across the data collection timeframe, the discussions were fairly consistent, with almost similar numbers of users, tweets, replies, or mentions being shared and discussed (see Table 1), but the interesting finding was the rate at which hashtags were used in those tweets. Even though a previous study found that users, especially professionals, used 12–23% of hashtags in their tweets (Bougie et al. 2011), our study found that 85.94% of users used hashtags, with two or more hashtags in their tweets, which is consistent with previous findings (Chae 2015). An average of five hashtags was used in the tweets, and a surprisingly high number for #motivation was found.

One of the interesting findings of this study was that many popular tweets were shared by people in the motivational speaking business, closely followed by people working in the psychological profession, and employees. Nearly all of the tweets were originally created tweets. It was also apparent that the most active users were the most visible users as well. This also showed that the majority of tweets were made by a very small percentage of users, which was backed by the centrality analysis of the network diagram. The first 9% of the users accounted for 56% of the tweets, which is consistent with previous studies (Zafar et al. 2020). 

In analyzing the sentiments of the tweets, the findings suggested that the overwhelming majority of the emotions were positive (as shown in Figure 4), which was consistent after standardizing the values of the sentiments. Good topic modeling performance was achieved in the study using the LDA model, which provided us with four distinct topics on which topic modeling could be carried out and the sentiment analysis could be modeled around. The four topics found were emotional intelligence, self-awareness, empathy, and motivation. The highest positive sentiment was found for motivation and the lowest for self-awareness; this is not saying much, as the difference between the highest and the lowest positive sentiment was 6.08% (highest = 94.61%; lowest = 88.53%). Negative and neutral sentiments were very low—almost marginal—when it came to their positive sentiment counterparts, suggesting that when people were talking about emotional intelligence and the topics related to it, the driving factors were positivity in their text. 

The findings showed that most of the people using the topics for their discussions of emotional intelligence were using Twitter to share their dealings in their personal, professional, and daily lives. These findings are consistent with previous studies which pertain to other fields (Stier et al. 2018; Wu et al. 2011; Kumar and Devi 2020). These were followed by EI advertisements, events, reports, and studies. As at the time of the study, the world was going through the distressful time of the pandemic, the platform of Twitter was being used as a tool to generate awareness about the advantages of being emotionally intelligent, which would explain the overwhelming positivity in the sentiments of the tweets, and the sparse but high user connectivity in the network analysis. Our findings on pandemic-related emotional discourse are partially aligned with similar studies (Rufai and Bunce 2020; Xue et al. 2020; Arora et al. 2021). This may also explain the disproportionately high diffusion of topics of motivation, empathy, self-awareness, and emotional intelligence, even though other prominent factors contribute to the understanding of emotional intelligence.

Analyzing these discussions on Twitter provides new avenues in understanding the affective undertones of the users talking about a soft skill such as emotional intelligence. This study also shows that studying social media adds an invaluable source of information for professionals or academic researchers, and this information can be used to make more informed decisions and effectively deal with the issues attached to it.

## 7. Study Implications

Twitter has been used by professionals in a myriad of ways (Vis 2013) and the findings of this study indicated that professionals having conversations on emotional intelligence are more conversational and information-focused than ideological. This is important for people pursuing careers in the domain of emotional intelligence because the business environment is dynamic and ever-changing, and it is adamant for these professionals to continually upgrade themselves with changes in the sources of knowledge on emotional intelligence.

It is also important to network and promote one’s skill set and expertise on online platforms (Holmberg and Thelwall 2014; Conway et al. 2013; Nason et al. 2015), not only for business professionals but also academic researchers. Professionals can take advantage of these metrics for learning, networking, and promotion. Sentiment analysis and topic modeling can provide a general consensus of the important topics that are being discussed, and what the overall sentiment of these discussions is. Similarly, network analysis can show the communication patterns amongst the users of interest and measure the intensity of these communications.

This can also be used to understand the type of people one is about to hire in an organizations. Because organizations are swiftly becoming global and competitive, social media could become one of the better tools for hiring professionals who are not only experts in their domain of expertise, but are also socially adept enough to be intelligent in their adaptive behavior. Mining of user information can allow for a better perspective on these potential candidates through their tweets, retweets, followers, or other engagements.

Some important research implications can also be derived from this study. The amount of data shared under each of the main hashtags of each topic was astonishing over the month-long study. They were also significantly diverse, as seen in the network topology. Emotional intelligence is also very relevant to other areas of research, such as behavior, personality, leadership, mental health, etc. This is also consistent with the findings of empirical studies in the area (Pathak et al. 2018; Shankar and Tewari 2021; Shearer 2018). The interdisciplinary nature of EI opens up new sets of diversions and cohesion for the future.

## 8. Limitations and Future Research

Professionals and academic researchers in the area of emotional intelligence must use Twitter as an important source of information. However, the EI world has been tediously slow in figuring out the importance of effectively using social media and the prominence it plays in developing research, academia, and the industry. One limitation of this study is the data collection. The timeframe of collecting the data was very small—less than a month—and thus, a longer time duration would allow for a better understanding of the contextual nature of the conversations regarding emotional intelligence. Another thing was the abundance of the words and hashtags of emotional intelligence. Further studies can look into multiple words or hashtags, preferably the topics identified in this study, which would further provide a more comprehensive picture of the domain of study. Another way could be to use the preferential keywords that could be identified using analytical techniques to mine tweets that cover other factors influencing emotional intelligence that have been identified by other prominent researchers (Goleman 1995; Mayer et al. 2000).

It is important to build an understanding of how social media plays a prominent role in emotional intelligence. This has to be built through academic investigations and practical applications. This can be achieved by creating guidelines that allow an understanding of the diverse practices of emotional intelligence and innovation in academia, and also through measuring investments into the performance of emotionally intelligent individuals and teams. There is an increasing need in the area of emotional intelligence, and big data and social media can provide an incentivized breakthrough.

## 9. Conclusions

In the present scenario of the dynamic environment, the study of emotional intelligence has increasingly become a significantly key research topic. The study of social media, big data, and natural language processing allows for analyzing, processing, and summarizing the information by using the affective and emotional spectrum of the content, which, in turn, allows measurement and analysis of the emotional competency of the users of online media. In the current domain of analysis of microblogging on emotional intelligence, there is an increasing need and demand for analyzing the role that emotional intelligence plays in users’ interactions. This study also analyzed the hidden features and characteristics of the users and their shared content, and opens avenues for establishing the connections between the users and their shared content.

This study aimed to explore the issues and evaluate the role that emotional intelligence plays in the users’ interactions on the microblogging giant Twitter, by analyzing the content through a framework that studies the descriptive, content, and network analytics (DA, CA, and NA). However, due to the limitations and other objective factors, there are still some areas that can be improved in future studies. For future work, researchers could conduct a more holistic emotional expression analysis that could comprehensively study the individual users’ characteristics, behavior, and personality, and how these can affect their judgments and decision-making. 

This is especially imperative for business organizations, as they are amongst the top contributors to the content on emotional intelligence on Twitter, and are amongst the most active and visible handles, which makes it clear that they are increasingly trying to use the platform and the theory of emotional intelligence to further better their business policies. This study thus opens up an avenue that can help both research academicians and industry professionals to increase their engagement and attention to further improve their workplace performance and outcomes.

## Figures and Tables

**Table 1:** Summary of the dataset.

Dates of Data Collection	14 February 2021–6 March 2021
Unique Users	22,895
No. of Tweets Collected	53,361
Maximum Number of Retweets on a Tweet	193
Maximum Number of Favourites	1801
Day with the Maximum Tweets	Monday
Hour with the Maximum Tweets	20:00
Date with the Maximum Account Creation by Users in the Data	25 February 2016

**Table 2:** Topic modeling and word analysis of the corpus.

Emotional Intelligence	Frequency	Self-Awareness	Frequency	Empathy	Frequency	Motivation	Frequency
emotional intelligence	1200	self-belief	1740	empathy	2872	motivation	49,530
eq	546	self-love	1566	care	1325	inspiration	13,383
emotions	368	well-being	398	self-care	1077	inspire	1328
intelligence	293	awareness	289	compassion	426	motivationalthoughts	1318
emotional health	67	self-awareness	232	understanding	178	determination	406
emotional quotient	48	belief	142	insight	140	inspired	280
ei	41	mental health	97	compassionate	41	commitment	95
emotionalwellbeing	22	workplacewellbeing	33	self-compassion	40	self-motivation	70

**Table 3:** Sentiment analysis of the tweet topics (in percentages).

Topics	Negative	Neutral	Positive
Emotional Intelligence	2.61%	4.76%	92.63%
Self-Awareness	2.95%	8.51%	88.53%
Empathy	2.16%	4.37%	93.47%
Motivation	1.22%	4.16%	94.61%

**Table 4:** List of high in-degree users.

Label	In-Degree	Out-Degree	Degree
iGotQ	24	19	43
Jayson Waller	19	17	36
Allan Beveridge	17	13	30
Dr. Gleb Tsipursky	15	11	26
Motivational Bytes	14	10	24
Waritha	12	12	24
ðŸ’‹	10	5	15
Thomas	9	10	19
Gemma Hawtin ðŸ’™	8	10	18
Patience Phillips ðŸ“š	7	15	22
â˜˜ï¸ keep pushing the limits â˜˜ï¸	7	9	16
The Obsidian Dragon	7	1	8
PeopleSkillsChat	4	3	7
STEERus	4	3	7
Money Making Conversations	4	8	12
Joaquin Salamanca	4	5	9
Super-A	4	7	11
Nikki Chopra	3	1	4
Debesh Choudhury	3	4	7
EQpassion	3	2	5

## Data Availability

All the extracted data and supplemental materials can be found on the corresponding author’s Github page at https://github.com/theshane007/EITwitterData.
